# Development of Microemulsion Containing *Alpinia galanga* Oil and Its Major Compounds: Enhancement of Antimicrobial Activities

**DOI:** 10.3390/pharmaceutics13020265

**Published:** 2021-02-15

**Authors:** Nattakanwadee Khumpirapang, Srikanjana Klayraung, Singkome Tima, Siriporn Okonogi

**Affiliations:** 1 Department of Pharmaceutical Chemistry and Pharmacognosy, Faculty of Pharmaceutical Sciences, Naresuan University, Phitsanulok 65000, Thailand; nattakanwadeek@nu.ac.th; 2 Program in Biotechnology, Faculty of Science, Maejo University, Chiang Mai 50290, Thailand; srikanja@mju.ac.th; 3 Department of Medical Technology, Faculty of Associated Medical Sciences, Chiang Mai University, Chiang Mai 50200, Thailand; singkome.tima@cmu.ac.th; 4 Research Center of Pharmaceutical Nanotechnology, Faculty of Pharmacy, Chiang Mai University, Chiang Mai 50200, Thailand; 5 Department of Pharmaceutical Sciences, Faculty of Pharmacy, Chiang Mai University, Chiang Mai 50200, Thailand

**Keywords:** antibacterial activity, antifungal activity, killing kinetics, essential oil, 1,8-cineole, methyl eugenol, drug delivery system, phase diagram

## Abstract

The aim of the present study was to develop a microemulsion (ME) containing *Alpinia galanga* oil (AGO), 1,8-cineole (C), or methyl eugenol (M) as an active pharmaceutical ingredient (API) for enhancing their antimicrobial activities. Agar diffusion, broth microdilution, and killing kinetics were used for antimicrobial evaluations. The ME composed of 30% API, 33.4% Tween 80, 16.6% ethanol, and 20% water appeared as translucent systems with droplet size and polydispersity index of 101.1 ± 1.3 nm and 0.3 ± 0.1, 80.9 ± 1.1 nm and 0.4 ± 0.1, and 96.6 ± 2.0 nm and 0.2 ± 0.1 for ME-AGO, ME-C, and ME-M, respectively. These ME formulations showed minimum bacterial concentrations of 3.91–31.25 µg/mL and 50% fungal inhibition concentrations of 1.83 ± 0.27–0.46 ± 0.13 µg/mL, 2–4 times stronger, and faster kinetic killing rate than their respective API alone. Keeping the ME formulations at 4 °C, 25 °C, and 40 °C for 12 weeks did not affect their activities against fungi and Gram-negative bacteria, but the high temperature of 40 °C decreased their activities against Gram-positive bacteria. It is concluded that ME is a promising delivery system for AGO and its major compounds to enhance their water miscibility and antimicrobial activities.

## 1. Introduction

Food safety is a highly important issue for consumers and food industries today. Contamination of different strains of bacteria such as *Bacillus cereus*, *Escherichia coli*, *Salmonella typhi*, *Shigella sonnei*, and *Staphylococcus aureus* can cause food deterioration and the bacteria can leach the toxic substances to humans [1,2]. In addition, fungal toxins from secondary metabolites produced by *Aspegillus flavus*, *Aspegillus niger*, and *Fusarium solani* are acutely and chronically toxic to humans and these toxins have also been specified as a potential biological weapon for food and water contamination [3]. Moreover, these toxins are carcinogenic compounds known to be produced in nature [4].

Rhizomes of *Alpinia galanga*, a member of the Zingiberaceae family, are edible and have been extensively used as condiments for flavoring in food and as a pharmaceutical ingredient (API) in traditional medicines for the treatment of stomachache and treating diarrhea [5]. It has been reported that the essential oils in rhizomes of *A. galanga* (AGO) have antimicrobial activities against several bacteria and fungi [6,7]. Our previous report described twenty-six chemical compounds, which represented 91.42% of the total compositions, that were obtained from AGO [8]. The major chemical compounds of AGO were 1,8-cineole (37.43%), 4-allylphenyl acetate (25.97%), and methyl eugenol (5.07%). AGO, 1,8-cineole, and methyl eugenol have been reported to have strong antibacterial and antifungal activities [9,10,11].

Essential oils from certain plants possess several important biological activities [12,13,14]. Among those activities, the antimicrobial activity of plant essential oils is one of the most interesting issues to be focused on because they might be useful as the natural antimicrobial alternatives to be used instead of the harmful chemical antimicrobial agents. The main causes of antimicrobial action of these essential oils are the destruction of the cytoplasmic membrane of the microorganisms by the oils or their major active compounds [15]. This can be achieved by accumulating in cell membranes, disturbing the cell membrane structure, and causing an increase of permeability of cell membrane leading to the leakage of intracellular constituents [16,17,18]. The extensive losses of these intracellular constituents can cause cell death [19,20].

Despite such high antimicrobial activity, the pharmaceutical role of AGO and its major chemical compounds has been restricted because of its low aqueous solubility, insufficient tissue absorption, and non-uniform distribution throughout the products which hamper their use as a therapeutic agent. Moreover, it has been reported that the antimicrobial activity of the essential oils can be reduced in the food systems due to the interfering in the interaction of essential oils with their target pathogens by food substances (lipids, proteins, proteases, etc.) [21]. To overcome these problems, a suitable drug delivery system is purposed. Microemulsion (ME) belongs to the group of colloidal drug delivery systems that consist of a certain amount of oil, water, surfactant, and/or cosurfactant [22]. ME is optically transparent, low viscosity, and thermodynamically stable [23]. ME has been used extensively in pharmaceutical applications. The advantage of ME is not only the low cost and ease of formulation, but also the improvement of bioavailability, aqueous solubility, and stability of lipophilic active pharmaceutical ingredients (APIs) [24]. It was reported that ME containing 5-Fluorouracil increased the flux of the drug 2–6-fold in comparison with the aqueous solution of the drug [25]. The stability of ascorbic acid was reported to be enhanced after entrapping in ME because the formulation could protect the drug from oxidation, and therefore its degradation rate was slower than in aqueous solutions [26]. The bioavailability of moxifloxacin entrapped in ME can be enhanced through its precorneal residence time and the drug release can be sustained [27]. Moreover, ME has been shown to enhance the biological activity of many natural compounds such as the wound healing effect of cinnamon oil [28] and the inhibition of cholinesterase activity of citrus oil [29].

The aim of this study was to develop ME formulations containing AGO (ME-AGO) and its major compounds, i.e., 1,8-cineole (ME-C) or methyl eugenol (ME-M), in order to enhance their aqueous solubility and biological activities against several strains of bacteria causing food deterioration and fungi producing harmful toxins to humans. In addition, physical and biological stabilities of the developed ME were also evaluated.

## 2. Materials and Methods

### 2.1. Materials and Microorganisms

AGO was extracted from freshly harvested *Alpinia galanga* rhizomes by hydro-distillation according to the method previously described [8]. 1,8-cineole and absolute ethanol were from Merck Millipore (Darmstadt, Germany). Tween 80 was from Merck (Hohenbrunn, Germany). Methyl eugenol was from Sigma-Aldrich (Copenhagen, Denmark). Gentamycin and nystatin were obtained from Sigma-Aldrich (St. Louis, MO, USA) and T.O. Pharma Co., Ltd. (Bangkok, Thailand), respectively. Mueller Hinton agar (MHA), trypticase soy agar (TSA), and potato dextrose agar (PDA) were from Himedia (Mumbai, India). Tryptic soy broth (TSB) was from Merck (Darmstadt, Germany). Double deionized water (DDW, 18 MΩcm) was prepared by a Milli-Q water purification system from Millipore Corp (Billerica, MA, USA). Other chemicals used were of the highest grade available.

Five bacterial strains, *Bacillus cereus* (DMST 5040), *Eecherichia coli* (DMST 4212), *Salmonella typhi* (DMST 5784), *Shigella sonnei* (DMST 561), and *Staphylococcus aureus* (DMST 8840), were obtained from the culture collections of the Department of Medical Sciences Thailand (DMST). The bacteria were kept on a TSA slant. Three fungal strains of *Aspergillus flavus* (TISTR 3130), *Aspergillus niger* (TISTR 3012), and *Fusarium solani* (TISTR 3412) were obtained from the Thailand Institute of Scientific and Technological Research (TISTR) Culture Collections, Bangkok, Thailand. These fungi were maintained on PDA.

### 2.2. Formulation of ME-AGO, ME-C, and ME-M

Pseudo ternary phase diagrams of AGO, 1,8-cineole, and methyl eugenol were constructed using a water titration method, in which AGO, 1,8-cineole, or methyl eugenol was used as one compartment and surfactant mixture (Smix) as another compartment in the phase diagram. To construct the phase diagram, Smix composed of Tween 80 and absolute ethanol at a weight ratio of 2:1 was firstly prepared. Subsequently, AGO, 1,8-cineole, or methyl eugenol was mixed with Smix at various weight ratios of 0:1, 1:9, 2:8, 3:7, 4:6, 5:5, 6:4, 7:3, 8:2, 9:1, and 1:0. The resulting mixtures were subsequently titrated with water under gentle agitation at room temperature. The mixtures were classified as ME when they appeared as clear liquids by visualization. The phase boundary was noted when the outer appearance of the mixtures transitioned from transparency to turbidity or vice versa. The pseudo ternary phase diagrams were drawn by OriginPro8 for Windows (OriginLab Corporation, Northampton, MA, USA). The experiment was done in triplicate.

From the pseudo ternary phase diagrams, the appropriate amount of each compartment was selected to formulate ME-AGO, ME-C, and ME-M by mixing the active compartment with Smix and water. The vortex mixer (Gemmy Industrial Co., Taipei, Taiwan) was used to mix the ingredients to homogenous preparations. Subsequently, the obtained ME formulations were transferred to closed-glass vials and kept for further use.

### 2.3. Characterization of ME-AGO, ME-C, and ME-M

The external appearance (phase separation, color, and turbidity) of each ME formulation was investigated by visualization. The droplet size and size distribution (polydispersity index; PdI) of each system was determined by photon correlation spectroscopy (PCS) using Zetasizer Nano ZS (Malvern Instruments Ltd., Malvern, UK) at a fixed angle of 173° with a temperature of 25 °C. At least ten measurements were performed in each experiment and the experiments were performed in triplicate.

### 2.4. Antibacterial Activity of ME-AGO, ME-C, and ME-M

Antibacterial activity of developed ME formulations in comparison with AGO and its main compounds was determined by disc agar diffusion, broth microdilution, and time-kill kinetics as follows.

#### 2.4.1. Agar Diffusion Analysis

A disc agar diffusion according to the National Committee for Clinical Laboratory Standards (NCCLS) standard method [30] was used for the preliminary determination of the antibacterial activity of the samples. The bacterial inoculum was obtained from overnight cultures in TSB at 37 °C and diluted by 0.85% *w/v* sodium chloride solution to have turbidity equivalent to 0.5 McFarland unit which was equivalent to the bacterial concentration of 10^8^ CFU/mL. The suspension of tested bacteria was swabbed on MHA containing 0.1% Tween 80. Sterile paper discs (6 mm in diameter) were separately added with 15 µL of each sample (30% *w*/*w* AGO in ethanol, 30% *w*/*w* 1,8-cineole in ethanol, 30% *w*/*w* methyl eugenol in ethanol, and their ME formulations (ME-AGO, ME-C, and ME-M) consisted of 30% *w*/*w* of AGO, 1,8-cineole, or methyl eugenol, respectively. The discs were put on the surface of the inoculated agar plates. The plates were incubated for 18–24 h at 37 °C. The results were recorded according to the diameter of inhibition zones against the tested bacteria. Gentamycin suspension (10 µg/mL) served as a positive control, while 70% *w*/*w* ethanol and ME without API (blank ME-API) were used as negative controls. The assay was done in triplicate.

#### 2.4.2. Broth Microdilution Analysis

The samples that showed antibacterial activity in the agar diffusion analysis were subjected to a broth microdilution analysis to determine a minimum inhibitory concentration (MIC) and/or a minimum bactericidal concentration (MBC). Two-fold dilutions of the samples were prepared in a 96-well plate to obtain the final concentrations of AGO, 1,8-cineole, or methyl eugenol over the range of 1.0–500.0 µg/mL in MHB (50 µL/each well). The bacterial strains were inoculated in TSB and incubated at 37 °C for 18–24 h. The obtained culture broth was adjusted to a turbidity of 0.5 McFarland unit which was equivalent to the bacterial concentration of 10^8^ CFU/mL. A volume of 50 µL bacterial suspension was added to each well to reach a final concentration of 10^6^ CFU/mL. The wells containing bacterial inoculum without any API served as a control. The plates were incubated at 37 °C for 18–24 h. The MIC is the lowest concentration of the test samples in the wells that showed neither visible bacterial growth nor turbidity after incubation. In the present study, MIC could not be determined because the turbidity of the samples occurred during the sample dilution process that interfered a visual determination of bacterial growth in the culture broths, and hindered the reading of MIC. However, MBC could be determined as follows. An exact volume of 10 µL of the bacterial culture from all wells was sub-cultured on MHA. The lowest sample concentration that completely killed initial bacteria, which was indicated by no visible growth on the MHA plate surface was considered the MBC. The experiment was performed in triplicate.

#### 2.4.3. Time-Killing Kinetic Study

In this experiment, 30% *w*/*w* AGO in ethanol, 30% *w*/*w* 1,8-cineole in ethanol, 30% *w*/*w* methyl eugenol in ethanol, ME-AGO, ME-C, and ME-M at the concentration of their MBC were used to investigated their bacterial killing rate. Ethanol (70% *w*/*w*) and blank ME-API were used as negative controls. *E. coli* was used as a model microorganism. The time-kill kinetic study was carried out using the method previously described [31] with some modification. Briefly, a stationary phase of *E. coli* was used to prepare a bacterial inoculum at a concentration of 10^8^ CFU/mL. A volume of 0.5 mL of *E. coli* inoculum was added into 4.5 mL of MHB. The mixture of bacterial culture and MHB containing each sample was vortexed for 15 sec and then incubated at 37 °C. Cell viability of *E. coli* in MHB containing the test samples and the controls over 20 min was investigated. Aliquots were interval taken at 0, 1, 3, 5, 10, 15, and 20 min. Bacterial viable counts on TSA were done and bacterial colonies were enumerated after incubation at 37 °C for 24 h. The killing kinetic profiles were plotted between Log colonies of the survival *E. coli* (CFU/mL) and time (min). The experiment was performed in triplicate.

### 2.5. Antifungal Activity of ME-AGO, ME-C, and ME-M

PDA containing 30% *w*/*w* AGO in ethanol, 30% *w*/*w* 1,8-cineole in ethanol, 30% *w*/*w* methyl eugenol in ethanol, ME-AGO, ME-C, and ME-M, at various final concentrations of active ingredient to cover a range of 0–5 µg/mL, were used for antifungal investigation. The 5 mm diameter mycelial plugs of each test culture were inoculated in the center of each PDA plate. The plates of fungal culture were incubated at room temperature for 7 days. Nystatin (10,000 units/mL) was used as a positive control, while 70% *w*/*w* ethanol and blank ME-API were used as negative controls. The experiment was performed in triplicate. Antifungal activity was determined using the antifungal index which was calculated by the following equation [32]

Antifungal index (%) = (1 − Da/Db) × 100(1)

where Da is the diameter of the fungal colony in the tested plate and Db is the diameter of the fungal colony in the control plate. For antifungal activity comparison among the samples, IC_50_ values (concentrations that produce 50% of the antifungal index) of the samples obtained from the dose-response curves based on measurement at different sample concentrations were used.

### 2.6. Stability Study

#### 2.6.1. Physical Stability

Outer appearances such as phase separation, color changes, and turbidity of the selected ME formulations kept at different storage temperatures of 4 °C, 25 °C, and 40 °C for 12 weeks were observed visually. In addition, the droplet size and size distribution of the fresh preparation samples was compared with the samples kept in the same manner. The experiment was done in triplicate.

#### 2.6.2. Antimicrobial Activity after Storage

Antibacterial and antifungal activities of the selected ME formulations that were kept at different storage temperatures of 4 °C, 25 °C, and 40 °C for 12 weeks were investigated using broth microdilution analysis and antifungal activity tests described in Section 2.4.2 and Section 2.5, respectively. The antibacterial and antifungal activities were expressed as MBC value and antifungal index, respectively. The experiment was performed in triplicate.

### 2.7. Statistical Analysis

All data are expressed as a mean ± SD of triplicate experiments. An independent *t*-test or a one-way ANOVA with Tukey’s post hoc test was used to analyze the droplet size and size distribution, antimicrobial activity, and physical stability of the ME formulations.

## 3. Results and Discussion

### 3.1. AGO and Its Chemical Compounds-Based ME Preparation and Characterization

**Figure 1:**
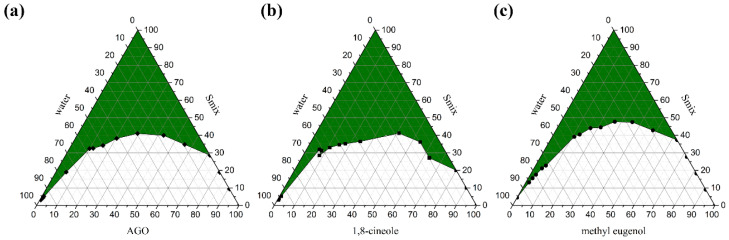
Pseudo ternary phase diagrams of *Alpinia galanga* oil (AGO) (**a**), 1,8-cineole (C) (**b**), and methyl eugenol (M) (**c**) using Tween 80 and absolute ethanol at a weight ratio of 2:1. The green area represents the monophasic area.

Pseudo ternary phase diagrams were created to identify an appropriate composition weight ratio of excipients; API, surfactant, cosurfactant, and water for ME formulations [14,33]. Three APIs; AGO, 1,8-cineole, and methyl eugenol, were used in the present study. It was found that different API affected the ME region in the phase diagrams as shown in Figure 1. The ME regions covered 43.88 ± 0.42%, 44.85 ± 0.29%, and 36.37 ± 0.27% of the overall area of the phase diagram of AGO, 1,8-cineole, and methyl eugenol as API, respectively. Moreover, the different types of surfactant and co-surfactants also affected the ME region in the phase diagrams as well as the ratio of Smix [29,34]. Our previous work found that Tween 80 and ethanol at a weight ratio of 2:1 was the best ratio and was an appropriate Smix for AGO in providing the largest monophasic area or ME area in the phase diagrams [35]. Therefore, Tween 80 and ethanol at this ratio were used as Smix in the present study. Tween 80 is an ethoxylated non-ionic surfactant and is popularly used in pharmaceutical products and research works due to its several beneficial properties such as non-ionic, non-toxic, and biocompatible, as well as inexpensive [36]. A wide range of pharmaceutical agents is available in formulations that contain polysorbate 80, including docetaxel, erythropoietin-stimulating agents, and various vaccines, etc. [37]. Thus, Tween 80 is safe for patients and consumers. Tween 80 can highly enhance the miscibility of oil and water by using certain co-surfactants like ethanol [38].

In the present study, nine ME formulations selected from the constructed phase diagram containing 20%, 25%, and 30% of each API with a constant water concentration of 20% were formulated. According to these different API concentrations, the number of 1, 2, and 3 were attached to the name of each ME, i.e., ME-AGO-1, ME-AGO-2, and ME-AGO-3 for the ME formulations containing 20%, 25%, and 30% AGO, respectively, ME-C-1, ME-C-2, and ME-C-3 for the ME formulations containing 20%, 25%, and 30% 1,8-cineole, respectively, and ME-M-1, ME-M-2, and ME-M-3 for the ME formulations containing 20%, 25%, and 30% methyl eugenol, respectively, as shown in Table 1. It was found that all ME formulations appeared as clear pale yellowish liquids without phase separation. The average droplet size of all ME-AGO, ME-C, and ME-M formulations were approximately 80–100, 50–80, and 40–100 nm, respectively, with PdI values of 0.3–0.5, 0.4–0.8, and 0.2–0.7, respectively. A lower PdI value indicates higher droplet size uniformity of the ME formulations. The results indicated that the droplet size of ME-AGO-3, ME-C- 3, and ME-M-3 was in a nano range with a narrow size distribution. Moreover, these formulations showed the relatively highest drug loading (30% API) among the developed formulations. This advantage would be useful when dose reduction must be applied. Therefore, these formulations were selected for further study on antimicrobial activity in comparison with their respective API.

### 3.2. Antimicrobial Activities ME-AGO, ME-C, and ME-M

#### 3.2.1. Antibacterial Activity

In this experiment, *B. cereus* and *S. aureus* were used as the representatives of Gram-positive bacteria whereas *E. coli*, *S. typhi*, and *S. sonnei* were used as the representatives of Gram-negative bacteria. The agar diffusion method is generally useful for the antimicrobial activity screening for both hydrophilic and lipophilic substances. However, for lipophilic substances, a cosolvent or surfactant might be needed to support the penetration of the substances into the agar medium. In the present study, as AGO and its main compounds, 1,8-cineole and methyl eugenol are lipophilic substances, they were mixed with ethanol before testing. The results, as shown in Table 2, demonstrated that AGO and its major compounds as well as their respective selected ME formulations possessed antibacterial activity against all tested bacteria. It was observed that the inhibition zones of the ME formulations were larger than that of the APIs alone. These results suggested that inhibition zone analysis is a good test for antimicrobial activity screening of these APIs and their microemulsions in the research work. Larger inhibition zones of the ME formulations suggested that microemulsion could provide high penetration enhancement of API into the agar medium. To confirm the antimicrobial activity of substances from the inhibition zone assay, the broth dilution test to determine MIC and MBC is generally performed. In the present study, there was interference from the turbidity of the samples during the dilution process, that the determination of MIC could not be possible. However, the MBC could be determined. It has been reported that using the inhibition zones from the agar plate method and the obtained MBC data can confirm the antibacterial activity of the samples [39,40].

The MBC values of the samples as shown in Table 3 confirmed the antibacterial activity of the test samples against the tested strains. It was previously reported, on antibacterial activity of the crude ethanolic extract of *A. galanga*, that the extract possessed high inhibitory effects against only Gram-positive bacteria [5,41]. It was reported that the main compounds of *A. galanga* ethanolic extract were the phenolic or derivatives of phenolic compounds, e.g., acetoxychavicol acetate and *p*-coumaryl diacetate [5]. Our present study showed that the essential oil of *A. galanga* having 1,8-cineole and methyl eugenol as the main components could inhibit both Gram-negative and Gram-positive bacteria. The essential oils of many plants having 1,8-cineole or methyl eugenol as the main component was previously revealed to have inhibitory effects against both Gram-negative and Gram-positive bacteria. Our results were in good agreement with the previous report [42].

Some previous studies reported that certain essential oils are more active against Gram-positive than Gram-negative bacteria because an outer membrane surrounding the cell wall of Gram-negative bacteria restricts the diffusion of hydrophobic compounds through its lipopolysaccharide covering [43,44]. However, some studies reported that Gram-positive bacteria are less susceptible to essential oils, e.g., the essential oil of *Mentha piperita* exhibited a greater antibacterial effect to *S. enteritidis* than for *L. monocytogenes* which were used as the representatives of Gram-positive and Gram-negative bacteria, respectively [45]. In addition, there were no significant differences in antibacterial properties of 50 tested commercially available essential oils against 25 genera between Gram-negative and Gram-positive bacteria [46]. It is, therefore, possible that individual compounds of essential oils achieved different degrees of activity against Gram-negative and Gram-positive bacteria [47]. Moreover, it is known that the quantitative and qualitative chemical compositions in the essential oils from the same plants can vary according to genetic factors, geography, physiology, and the harvest period of the plants [48], thereby causing variability in the degree of susceptibility of Gram-negative and Gram-positive bacteria.

Considering AGO, previous studies reported that AGO had a broad range of antimicrobial activity against a variety of bacteria and fungi [5,49]. Normally, the major groups of principal compounds that make essential oils effective antimicrobials include flavonoids, eugenol, terpenes, thymol, citral, carvacrol, and saponin [21]. By chemical structure, 1,8-cineole and methyl eugenol can be categorized into the group of terpenes and eugenol derivatives, respectively. Therefore, these compounds are considered major antibacterial components of AGO. From the MBC results as in Table 3, it was noted that methyl eugenol possessed a greater activity than AGO and 1,8-cineole, respectively, in inhibition against the Gram-positive tested bacteria. However, for Gram-negative bacteria, methyl eugenol showed the same inhibitory potential as AGO against *S. typhi* and *S. sonnei* but less activity against *E. coli*. The strong activity against *E. coli* might be due to the synergistic effect. The minor compounds in AGO might be critical to the bactericidal effect and have a synergistic effect on the major compounds existing in AGO. In addition, some studies have reported that the whole essential oil exhibited greater antibacterial activity than major or minor compounds alone [50,51]. Considering the MBC data of the tested bacteria, our results suggested that Gram-negative bacteria were more slightly sensitive to AGO and its ME formulation than Gram-positive bacteria.

Interestingly, the selected ME formulations of all API showed significantly higher antibacterial activity against the tested bacteria than their respective API (*p* < 0.05), e.g., the MBC values of AGO against *B. cereus* (15.63 µg/mL) and *S. sonnei* (7.81 µg/mL) were two times higher than that of ME-AGO-3 (7.81 and 3.91 µg/mL, respectively) and the MBC values of 1,8-cineole against *B. cereus* (31.25 µg/mL), *S. typhi* (31.25 µg/mL), and *S. sonnei* (31.25 µg/mL) were four times higher than that of ME-C-3 (7.81 µg/mL). These results indicated that the bactericidal effects of ME-AGO-3 and ME-C-3 were two and four times stronger than that of AGO and 1,8-cineole alone, respectively. The MBC result of methyl eugenol and its ME formulation were in accordance with that of AGO and 1,8-cineole. These results demonstrated the potential of the developed ME formulations to enhance the antibacterial activity of AGO, 1,8-cineole, and methyl eugenol.

**Figure 2:**
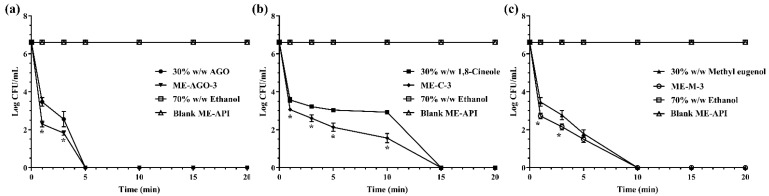
Killing kinetics of AGO and its ME (**a**), 1,8-cineole and its ME (**b**), methyl eugenol and its ME (**c**) against *Escherichia coli*. Asterisk (*) indicates significant differences between groups (*p* < 0.05).

Time-killing kinetic analysis was normally used for the determination of rapidity and duration of antibacterial activity [52]. The survival curve was plotted between the number of viable cells remaining in the broth after exposure to the tested treatments and time. In the present study, *E. coli* was used as the model microorganism, due to its susceptibility to the tested samples and being a common foodborne pathogen. It generally contaminates food during production and processing as well as during storage and transport before consumption [53,54]. In addition, *E. coli* is an important pathogenic bacterium that causes diarrhea in humans [55]. Figure 2 demonstrates the killing kinetics of the test samples against *E. coli* within 15 min whereas 70% *w*/*w* ethanol and blank ME-API that contain the same amount of Tween 80 in those formulations did not show killing activity throughout the test period of 20 min, indicating that Tween 80 had no antibacterial activity. Our result was in good agreement with previous work reported by the other group that the broth with Tween 80 alone did not inhibit the growth of the test bacteria [28]. The results demonstrated that AGO and its ME had significantly faster killing ability than methyl eugenol and its ME, and 1,8-cineole and its ME, respectively. The times required for complete killing *E. coli* were 5, 10, and 15 min for AGO and its ME, methyl eugenol and its ME, and 1,8-cineole and its ME, respectively. In addition, it was noted that before the complete killing, the killing rate of ME formulations of all API was faster than their API alone. Such rapid killing ability of ME formulations is indicative of a direct attack on the structural integrity of the cell, which can cause membrane structure dysfunction [56].

#### 3.2.2. Antifungal Activity

The results of this experiment as the antifungal index could be plotted versus sample concentrations. According to these plots, IC_50_ values of the test samples could be determined. From the obtained IC_50_ values of the test samples, as shown in Table 4, it was found that ME-C-3 had inhibitory effects against *F. solani* (IC_50_ = 1.83 ± 0.27) whereas 1,8-cineole did not show antifungal activity. It was previously reported that 1,8-cineole possessed antifungal activity [57]. However, Vilela et al. [58] reported that the 1,8-cineole showed antifungal effects only at a high concentration. The lack of activity of 1,8-cineole against *F. solani* in the present study might be due to the insufficient concentration of 1,8-cineole as well as lower ability to enter the cells. However, the antifungal activity of ME-C-3 confirmed the antifungal of 1,8-cineole even in low concentration. This result showed the potential of ME on drug enhancement into the fungal cells. Among the test API, methyl eugenol exhibited the highest antifungal activity against all tested fungal species. These data were in agreement with the previous study that reported the fungicidal activity of methyl eugenol against several fungi including *Aspergillus* species [59,60]. These previous works also reported that methyl eugenol had the ability to damage the plasma membrane by structural and functional integrity, including sterol biosynthesis. Furthermore, the fungicidal activity of ME-AGO and ME-M against all tested fungal species were one to four times stronger than that of AGO and methyl eugenol alone, confirming the effectiveness of ME on enhancing the loaded antifungal agent into the fungal cells.

### 3.3. Stability Study

#### 3.3.1. Physical Stability

**Figure 3:**
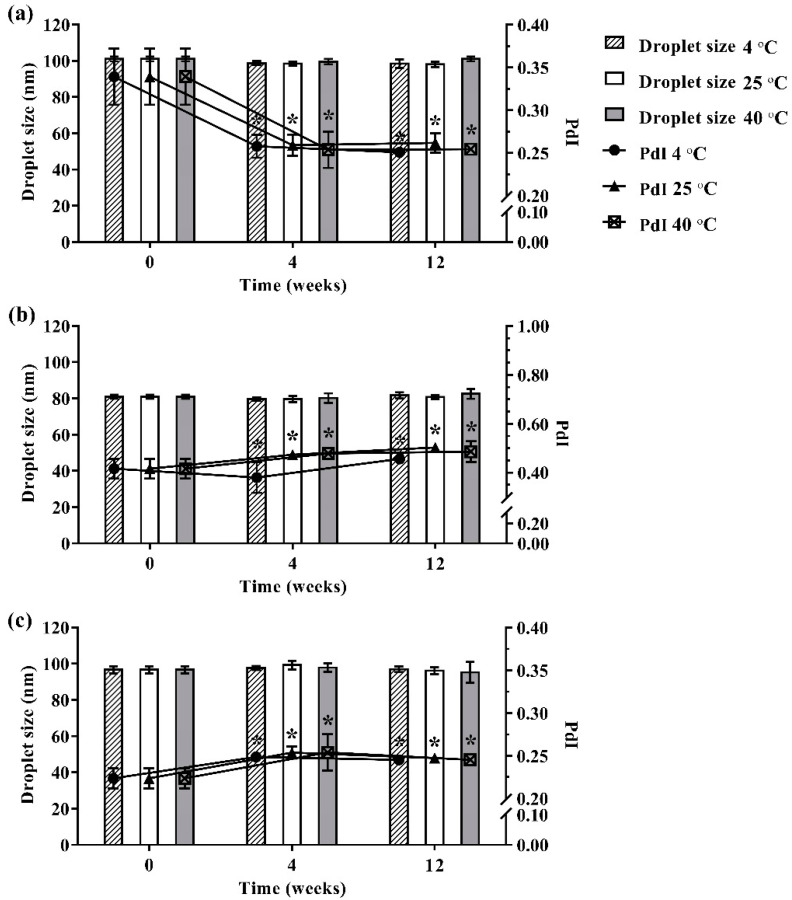
Physical stability of ME-AGO-3 (**a**), ME-C-3 (**b**), and ME-M-3 (**c**). Data are presented as mean ± SD. Asterisks (*) demonstrate the significant difference of droplet size between ME formulations kept at different storage temperatures (4 °C, 25 °C, and 40 °C) and freshly prepared of those formulation (*p* < 0.05).

During 12 weeks of storage, no phase separation, color change, or turbidity occurred in the developed ME formulations that were kept at different storage temperatures (4 °C, 25 °C, and 40 °C). Moreover, no ME formulation showed any significant difference in droplet size as shown in Figure 3. However, size distribution was slightly changed in the ME formulations during 12 weeks of storage. The results indicated that storage time and temperature did not affect the droplet size of the developed ME formulation but slightly influenced size distribution.

#### 3.3.2. Antimicrobial Activity after Storage

**Figure 4:**
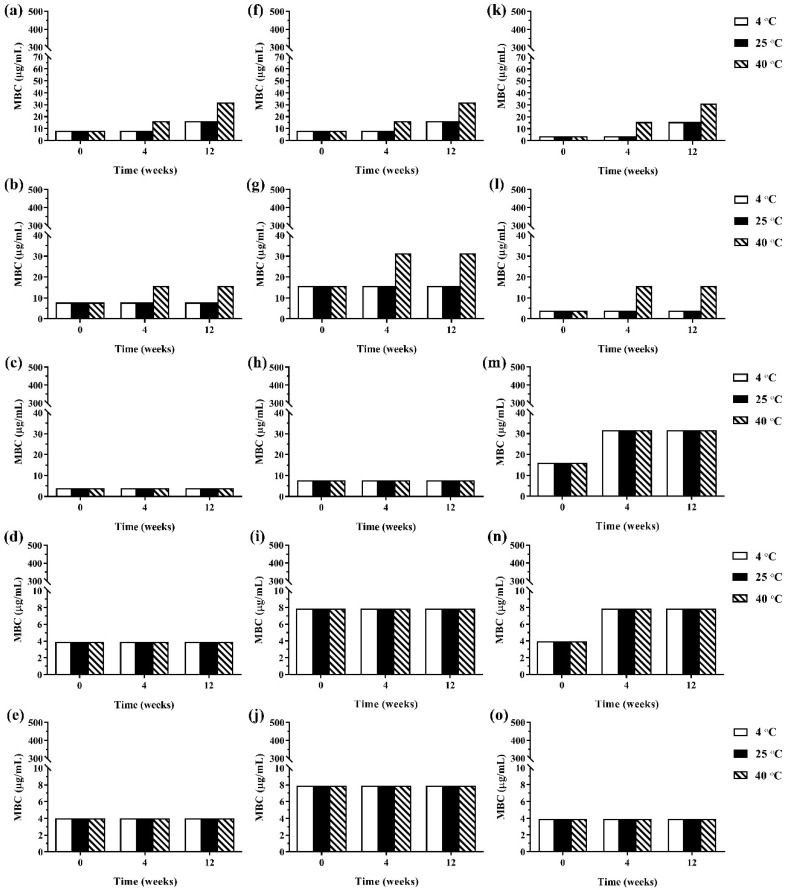
The minimum bactericidal concentrations (MBCs) values of ME formulations kept at different storage temperatures (4 °C, 25 °C, and 40 °C); ME-AGO-3 against *B. cereus* (**a**), *S. aureus* (**b**), *E. coli* (**c**), *S. typhi* (**d**), and *S. sonnei* (**e**), ME-C-3 against *B. cereus* (**f**), *S. aureus* (**g**), *E. coli* (**h**), *S. typhi* (**i**), and *S. sonnei* (**j**), and ME-M-3 against *B. cereus* (**k**), *S. aureus* (**l**), *E. coli* (**m**), *S. typhi* (**n**), and *S. sonnei* (**o**).

**Figure 5:**
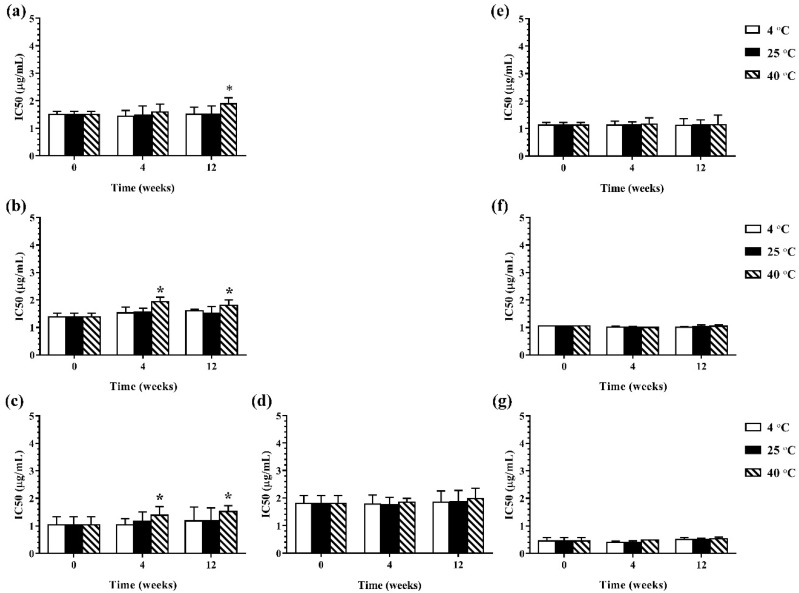
The IC_50_ values of ME formulations kept at different storage temperatures (4 °C, 25 °C, and 40 °C); ME-AGO-3 against *A. flavus* (**a**), *A. niger* (**b**), and *F. solani* (**c**), ME-C-3 against *F. solani* (**d**) and ME-M-3 against *A. flavus* (**e**), *A. niger* (**f**), and *F. solani* (**g**). Asterisk (*) indicates significant differences between groups (*p* < 0.05).

During 12 weeks of storage, there was no change in antimicrobial activity of the developed ME formulations kept at 4 °C and 25 °C. However, the ME formulations that were kept at 40 °C showed some changes in antibacterial and antifungal activities against certain microorganisms. The results of the antibacterial activity of the developed ME formulations after storage are shown in Figure 4. It was found that keeping at 40 °C, the MBC values of the ME formulations against Gram-positive bacteria (*B. cereus* and *S. aureus*) were increased compared to the freshly prepared samples whereas the MBC values of these formulations kept at all test storage temperatures against Gram-negative bacteria (*E. coli*, *S. typhi*, and *S. sonnei*) showed no change. These results suggested that the storage temperature played no influence on the antibacterial activity of the ME formulations against Gram-negative bacteria. For antifungal activity, the results are shown in Figure 5. It was found that during 12 weeks of storage, there was no change of IC_50_ values of ME-M-3 that was kept at different storage temperatures (4 °C, 25 °C, and 40 °C) against all tested fungal species (*A. flavus*, *A. niger*, and *F. solani*). IC_50_ values were slightly increased but not significant in ME-C kept at 40 °C. These results indicated that storage temperature did not significantly affect the antifungal activity of the developed ME formulations kept for 12 weeks.

From the results of the present study, the advantages of the produced ME are obvious. The developed ME-API formulations can solubilize the API and provide uniform distribution of the API throughout the products. They have the ability to retain the APIs from evaporation and enable the gradual release of API to achieve long-term efficacy during shelf-storage. The most suitable formulated ME-API can significantly enhance the antimicrobial activities of its respective API alone. The higher antimicrobial activity of the ME-API against the tested microorganisms is considered to be due to the formulation of nanodroplets of ME that increase the surface tension and thereby force themselves to merge with the lipophilic components in the microbial cell membrane [61]. Furthermore, the water in the external phase of the ME-API can be tightly bound to the internal oily phase and not available to the microorganisms for their growth [62]. The ingredients used in the ME-API formulations are safe for humans. Thus, the ME-API can be used as a promising natural alternative to chemical preservatives to prevent the growth of bacteria or fungi in liquid or semisolid forms of food and pharmaceutical products such as solutions, lotions, creams, and gels or even to coat solid materials whose surface is also consumed.

## 4. Conclusions

The ME formulations of AGO and its two major compounds, 1,8-cineole and methyl eugenol as API, can be developed using a pseudo ternary phase diagram. The most suitable ME-API comprises 30% of API, 50% of Tween 80-ethanol mixture (2:1 weight ratio), and water. The developed ME-API possesses significantly stronger antimicrobial activities than their respective APIs alone. The developed formulations are stable after storage at low temperatures of 4 °C and 25 °C. It can be concluded that the developed ME formulations are promising delivery systems for AGO and its major compounds to enhance their antimicrobial activities as well as decrease the use of alcohol in the formulations.

## Figures and Tables

**Table 1:** Composition of microemulsion (ME) formulations (% *w*/*w*).

Formulation	API		Smix	Water	Size (nm)	PdI
ME-AGO-1	AGO	20	60	20	85.7 ± 0.5 a	0.5 ± 0.1 a
ME-AGO-2		25	55	20	93.10 ± 1.7 b	0.5 ± 0.1 a
ME-AGO-3		30	50	20	101.1 ± 1.3 c	0.3 ± 0.1 b
ME-C-1	1,8-cineole	20	60	20	55.7 ± 1.8 a	0.8 ± 0.1 a
ME-C-2		25	55	20	74.4 ± 2.6 b	0.4 ± 0.1 b
ME-C-3		30	50	20	80.9 ± 1.1 c	0.4 ± 0.1 b
ME-M-1	methyl eugenol	20	60	20	41.8 ± 4.6 a	0.7 ± 0.1 a
ME-M-2		25	55	20	74.9 ± 0.1 b	0.2 ± 0.1 b
ME-M-3		30	50	20	96.6 ± 2.0 b	0.2 ± 0.1 b

Data are presented as mean ± SD of three replicates. Different letters indicate significant difference between treatment groups (*p* < 0.05).

**Table 2:** Antibacterial activity of 30% *w*/*w* AGO in ethanol, 30% *w*/*w* 1,8-cineole in ethanol, 30% *w*/*w* methyl eugenol in ethanol, and their microemulsion (ME) formulation against selected foodborne pathogens (*n* = 3).

Samples	Diameter of Inhibition Zone * (mm)				
	*B. cereus*	*S. aureus*	*E. coli*	*S. typhi*	*S. sonnei*
AGO	7.4 ± 0.2 a	7.8 ± 0.2 a	8.4 ± 0.3 a	8.1 ± 0.2 a	7.0 ± 0.1 a
1,8-cineole	7.2 ± 0.3 a	7.5 ± 0.5 a	6.1 ± 0.2 a	7.2 ± 0.1 a	7.6 ± 0.4 a
Methyl eugenol	7.7 ± 0.3 a	8.2 ± 0.1 a	7.5 ± 0.3 a	8.2 ± 0.3 a	7.1 ± 0.2 a
ME-AGO-3	10.4 ± 0.7 b	11.4 ± 0.2 b	8.2 ± 0.3 b	7.6 ± 0.5 a	9.2 ± 0.6 a,b
ME-C-3	11.9 ± 0.3 b	12.4 ± 0.5 c	8.8 ± 0.2 c	8.9 ± 0.4 b	10.5 ± 0.5 b
ME-M-3	10.4 ± 1.0 b	11.3 ± 0.2 b	8.1 ± 0.4 b	8.1 ± 0.2 a	9.5 ± 0.6 a,b
Gentamycin	17.1 ± 2.1 c	22.1 ± 0.5 d	21.2 ± 0.4 d	22.6 ± 0.3 c	23.1 ± 0.2 c
Ethanol	NZ	NZ	NZ	NZ	NZ
Blank ME-API	NZ	NZ	NZ	NZ	NZ

NZ = No zone. * Data are presented as mean ± SD of three replicates. Different letters indicate significant difference between treatments (*p* < 0.05).

**Table 3:** Minimum bactericidal concentrations (MBCs) (µg/mL) of 30% *w*/*w* AGO in ethanol, 30% *w*/*w* 1,8-cineole in ethanol, 30% *w*/*w* methyl eugenol in ethanol, and their ME formulation (*n* = 3).

Samples	Foodborne Bacteria				
	*B. cereus*	*S. aureus*	*E. coli*	*S. typhi*	*S. sonnei*
AGO	15.63	15.63	7.81	7.81	7.81
1,8-cineole	31.25	31.25	15.63	31.25	31.25
Methyl eugenol	7.81	7.81	31.25	7.81	7.81
ME-AGO-3	7.81	15.63	7.81	7.81	3.91
ME-C-3	7.81	31.25	15.63	7.81	7.81
ME-M-3	15.63	15.63	15.63	3.91	7.81
Gentamycin	0.13	0.13	0.49	0.13	0.13
Ethanol	125	250	250	125	125
Blank ME-API	250	>500	>500	250	>500

**Table 4:** IC_50_ values (µg/mL) of 30% *w*/*w* AGO in ethanol, 30% *w*/*w* 1,8-cineole in ethanol, 30% *w*/*w* methyl eugenol in ethanol, and their ME formulation (*n* = 3).

Samples	Fungal Species *		
	*A. flavus*	*A. niger*	*F. solani*
AGO	2.47 ± 0.05 a	2.01 ± 0.03 a	3.24 ± 0.10 a
1,8-cineole	>5	>5	>5
Methyl eugenol	2.02 ± 0.03 b	1.88 ± 0.01 a	2.00 ± 0.07 b
ME-AGO-3	1.53 ± 0.09 c	1.38 ± 0.14 b	1.04 ± 0.29 c
ME-C-3	>5	>5	1.83 ± 0.27 b
ME-M-3	1.15 ± 0.07 d	1.05 ± 0.01 c	0.46 ± 0.13 d
Nystatin	1.22 ± 0.09 d	1.30 ± 0.08 b	1.04 ± 0.20 c
Ethanol	NA	NA	NA
Blank ME-API	NA	NA	NA

NA = No activity. * Data are presented as mean ± SD of three replicates. Different letters indicate significant difference between treatments (*p* < 0.05).

## Data Availability

All data are available upon request.
